# The mental health of laboratory and rehabilitation specialists during COVID-19: A rapid review

**DOI:** 10.3934/publichealth.2023006

**Published:** 2023-02-17

**Authors:** Liam Ishaky, Myuri Sivanthan, Behdin Nowrouzi-Kia, Andrew Papadopoulos, Basem Gohar

**Affiliations:** 1 Department of Population Medicine, University of Guelph, 50 Stone Rd E. Guelph, ON, N1G 2W1, Canada; 2 Department of Occupational Science & Occupational Therapy, University of Toronto, 27 King's College Cir, Toronto, ON, M5S 1A1, Canada; 3 Centre for Research in Occupational Safety & Health, Laurentian University, 935 Ramsey Lake Rd, Sudbury, ON, P3E 2C6, Canada

**Keywords:** COVID-19, mental health, medical laboratory professionals, occupational therapists, physical therapists, rapid review

## Abstract

**Backgrounds:**

Healthcare workers have experienced considerable stress and burnout during the COVID-19 pandemic. Among these healthcare workers are medical laboratory professionals and rehabilitation specialists, specifically, occupational therapists, and physical therapists, who all perform critical services for the functioning of a healthcare system.

**Purpose:**

This rapid review examined the impact of the pandemic on the mental health of medical laboratory professionals (MLPs), occupational therapists (OTs) and physical therapists (PTs) and identified gaps in the research necessary to understand the impact of the pandemic on these healthcare workers.

**Methods:**

We systematically searched “mental health” among MLPs, OTs and PTs using three databases (PsycINFO, MEDLINE, and CINAHL).

**Results:**

Our search yielded 8887 articles, 16 of which met our criteria. Our results revealed poor mental health among all occupational groups, including burnout, depression, and anxiety. Notably, MLPs reported feeling forgotten and unappreciated compared to other healthcare groups. In general, there is a dearth of literature on the mental health of these occupational groups before and during the pandemic; therefore, unique stressors are not yet uncovered.

**Conclusions:**

Our results highlight poor mental health outcomes for these occupational groups despite the dearth of research. In addition to more research among these groups, we recommend that policymakers focus on improving workplace cultures and embed more intrinsic incentives to improve job retention and reduce staff shortage. In future emergencies, providing timely and accurate health information to healthcare workers is imperative, which could also help reduce poor mental health outcomes.

## 1. Introduction

The COVID-19 pandemic caused significant disruptions to the healthcare system worldwide. Despite pandemic mitigation strategies, including preventative measures to reduce mortality, such as vaccination and social distancing [1], the pandemic has affected healthcare workers' mental health extensively [2]. Results from recent studies suggest that healthcare workers have been experiencing poor mental health outcomes during the pandemic, including anxiety, depression, work stress, and trauma-related symptoms [2]–[6]. Their ability to recover outside the workplace was also impaired by markedly higher than-normal reports of insomnia and sleep difficulties [6]. In addition to their high work demand and poor mental health during the pandemic, healthcare workers face new challenges, including cognitive dissonance related to their exposure risks and perceptions of vaccinations [7].

While there are commonly known healthcare professions within the scientific literature, such as nurses, others are less known despite their critical roles, especially during the pandemic. Among these healthcare workers are medical laboratory professionals (MLPs) and rehabilitation specialists such as occupational therapists (OTs) and physical therapists (PTs). Throughout the pandemic, MLPs have continued to perform laboratory services and were tasked with additional duties such as analysis of COVID-19 tests, which are vital for the operations of a healthcare network [8]. OTs provide rehabilitation services that make discharges from care centers safer for patients and more efficient for hospitals, allowing needed beds to become freed up for incoming patients [9],[10]. Furthermore, OTs' physical and cognitive rehabilitation services reduced hospital readmission rates [11]. Similarly, PTs provided rehabilitation services, specifically pulmonary rehabilitation, to COVID-19 patients, improving dyspnea and reducing complications and disability [12].

Like other healthcare workers, MLPs, OTs, and PTs have all experienced elevated stress during the COVID-19 pandemic [8],[13],[14]. In addition, studies before the pandemic revealed that PTs and OTs were experiencing poor mental health outcomes, such as burnout [15]–[17]. For example, Śliwiński et al. (2014) found that 63.5% of PTs surveyed had at least moderate levels of burnout [15]. Additionally, Gupta et al. (2012) reported significant levels of emotional exhaustion, cynicism, and low perceived professional efficacy among OTs [18]. Notably, we could not find any studies in the extant literature examining the mental health of MLPs before the pandemic. With some evidence suggesting poor mental health outcomes for PTs and OTs before the pandemic, these groups are ideally situated to be grouped in this rapid review as they are underrepresented in the literature based on our search. Recognizing the dearth of literature among MLPs and rehabilitation specialists in general and the results from recent studies of other healthcare workers signaling poorer-than-usual mental health outcomes [2]–[7], the objective of this rapid review was to examine the mental health of OTs, PTs, and MLPs during the pandemic.

## 2. Materials and methods

### 2.1. Sample and data

As this is a review article, ethics approval was not required. We utilized the Cochrane review guidelines for rapid reviews to create this review article. We consulted a librarian scientist to assist in designing our search strategy for this review. In consultation with the librarian and the research team, we selected MEDLINE, CINAHL, and PsycINFO as the databases for our search. We created a list of search terms and subject headings through cursory searches and suggestions from the librarian. From these search terms, we created Boolean phrases and applied them to the three databases to examine the mental health of MLPs, OTs, and PTs throughout the pandemic. Please see Supplementary Materials for the complete search strategy. MLPs, OTs, and PTs were the population of interest in this study. We excluded articles that pooled MLPs, OTs, or PTs with other healthcare worker groups.

### 2.2. Measures of variables

We broadly defined “mental health” as a state of emotional and psychological well-being [19]. Mental health during the pandemic was the outcome of interest in this review. Additionally, this review examined articles between March 2020 and May 2022. We included studies published as primary and secondary research articles that were quantitative and qualitative studies. We limited our selection of studies to those written in English.

### 2.3. Data synthesis procedure

We used the software Covidence for all screening and data extraction in this rapid review [20]. Prior to reviewing articles, we performed a pilot exercise with 50 articles relevant to the rapid review between two reviewers (L.I. & M.S.), to determine consistency between raters and the relevance of the articles based on the search terms, which helped us fine-tune the selection criteria before the screening process. We then performed the initial abstract and title screen, full-text screening, and data extraction. Data extraction included: First author and year of publication, study information including origin, population, research design and objectives, and results pertinent to the groups of interest (Table 1).

## 3. Results

**Figure 1.**
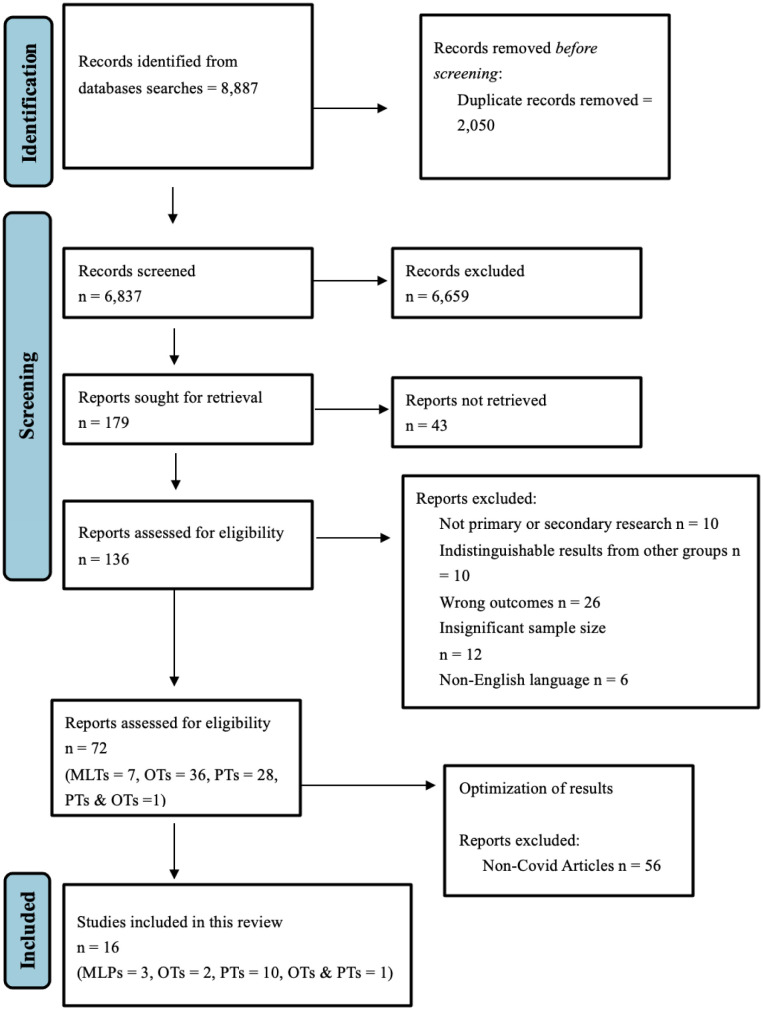
PRISMA Chart depicting article screening process [22].

Our initial search yielded 8887 articles. After removing duplicates and screening, 72 were analyzed. Next, we excluded studies that did not examine the impact of COVID-19 on the mental health of MLPs, OTs, and PTs, leading to 16 articles. Of these studies, three articles were related to MLPs, three were related to OTs, and 10 were related to PTs. One study examined several healthcare groups, including OTs and PTs [21]. The PRISMA chart (Figure 1) reflects these findings [22]. We utilized twelve cross-sectional studies, three qualitative studies, and one mixed-method study. Of the 16 included studies, 41% were conducted in Europe (Germany, Cyprus, Portugal, Spain, and Poland), and 23.5% were conducted in Asia (Japan, China, and South Korea). Additionally, three studies were conducted in North America (Canada and The United States), two studies were conducted in South America (Brazil), and one study was conducted in Africa (South Africa).

We found two cross-sectional studies and one qualitative study examining MLPs [8],[23],[24] (Table 1). Studies from Canada and Japan identified that 72.3% and 13.3% of MLPs experienced burnout, respectively [23],[24]. The Canadian study revealed that being over 50, having high quantitative demand, high work pace, high job insecurity, and high work-life conflict were positively correlated with burnout [23]. Moreover, the Japanese study revealed that MLPs had 6.1 greater odds of experiencing burnout than physicians (95%CI 2.0–18.5, *p* = 0.002) [24]. Also, a Canadian qualitative study revealed that staff shortage caused significant stress among MLPs [8]. Participants also reported feeling unappreciated and forgotten and experiencing a poor work environment that has been worsened because of the pandemic.

**Table 1.** Summary of studies examining the mental health of MLPs during COVID-19 (n = 3).

First author, year	Study information	Results
Gohar, 2022 [8]	Country: Canada Design: qualitative Objective: understand COVID-related stressors.	- The pandemic contributed to a notable staff shortage, causing increased work and stress, including moral distress. - Feeling unappreciated and forgotten. - Poor work environment exacerbated by the pandemic.
Nowrouzi-Kia, 2022 [23]	Country: Canada Design: Cross-sectional Objective: examine correlates of burnout during the pandemic.	- Almost 75% of participants reported burnout. - Over half the sample had worse job satisfaction than before the pandemic. - Age (older), education level (lower), quantitative demands (higher), work pace (higher), job insecurity (higher), work-life conflict (higher), job satisfaction (lower) and self-rated health (lower) were all statistically correlated with burnout.
*Matsuo, 2020 [24]	Country: Japan Design: Cross-sectional Objective: examine correlates of burnout during the pandemic.	- 13.3% experienced burnout. - MLPs had greater odds of burnout than physicians (referent group).

*Note: *Several occupations, including MLPs.

Three cross-sectional studies examined the mental health of OTs during the pandemic [21],[25],[26]. In one study that examined the mental health of various healthcare groups, OTs reported a perceived increase in overall burden during the pandemic. Participants were asked to retrospectively compare their level of burden before and during the pandemic. Specifically, 45.5% experienced a very low or low overall burden, while only 15.2% experienced a high or very high burden before the pandemic. During the pandemic, only 13% reported a low or very low burden while 50.5% experienced a high or very high burden [21]. Additional analyses were included in the study; however, they were combined with other occupational groups and, therefore, could not be interpreted for this study. In a Japanese study, the researchers examined the psychological impact of COVID-19 on OTs in regions with specific COVID-19 advisements (e.g., restrictions) and without advisements [25]. In regions with specific caution, avoiding face-to-face conversation increased the risk of anxiety, while decreased workload reduced the risk of anxiety. In regions without specific COVID-19 caution, those who did not receive sufficient information on COVID-19, experienced a high workload, or avoided face-to-face conversation had an increased risk of anxiety. The high workload increased the risk of depression among those in regions under specific caution. In regions without caution, increased workload, efforts not to transmit the virus, decreased contact with friends, and avoiding face-to-face conversations increase the risk of depression. Alternatively, those who had sufficient information provided to them about COVID-19 had a lower risk of depression. A higher workload increased the risk of insomnia among those under specific caution, and sufficient information provided about COVID-19 reduced the risk of insomnia [25]. Finally, a Chinese study examined the role of social support on depression among OTs working in Hong Kong during the pandemic [26]. The results revealed that perceived social support is a protective factor against depression.

**Table 2.** Summary of studies examining the mental health of OTs during COVID-19 (n = 3).

First author, year	Study information	Results
*Borusiak, 2022 [21]	Country: Germany Design: Cross-sectional Objective: Examine overall burden and mental health of several healthcare providers during the pandemic.	- Retrospectively, OTs and PTs reported an increase in overall burden during the pandemic compared to before the pandemic.
Ishioka, 2021 [25]	Country: Japan Design: Cross-sectional Objective: Investigate psychological impact of COVID-19 on OTs.	1.a. Regions under specific caution and risk of anxiety: - Avoiding face-to-face conversations increased the risk. - Decreased workload reduced the risk. 1.b. Regions without specific caution and risk anxiety: - Insufficient information provision about COVID-19, increased workload and avoiding face-to-face conversations increased the risk. 2.a. Regions under specific caution and risk of depression: - Increased workload increased the risk. 2.b. Regions without specific caution and risk of depression: - Increased workload, efforts not to transmit the virus to others, decreased contact with friends and avoiding face-to-face conversation increased the risk. - Sufficient information provision about COVID-19 reduced the risk. 3.a. Regions under specific caution and risk of insomnia: - Increased workload and daily step count increased the risk. 3.b. Regions without specific caution and risk of insomnia: - Sufficient information provision about COVID-19 reduced the risk.
Chan, 2022 [26]	Country: China Design: Cross-sectional Objective: Examine the role of perceived support on depression during the pandemic.	- Increased social support served as a protective factor, reducing the risk of depression.

*Note: *Includes OTs and PTs.

Our search revealed one study that examined OTs and PTs among other healthcare providers [21]. Additional seven cross-sectional studies [14],[27]–[32], one mixed-method [33] and two qualitative studies [34],[35] were retrieved all of which were scoped exclusively to PTs. Similar to OTs, PTs' overall burden increased during the pandemic. For instance, before the pandemic, almost 60% of PTs reported low or very low overall burden, and only 7.2% reported high or very high burden. In contrast, only 15.9% reported low or very low burden while almost 45% reported a high or very high burden during the pandemic [21].

Duarte et al. (2022) examined the factors associated with PTs' stress perception during the pandemic [27]. Results revealed that those working with COVID-19 patients had higher stress levels than those without. Furthermore, factors including sex (female), younger age, previous diagnosis of depressive or anxiety disorder, worsening in sleep patterns, a large reduction in family income, housework, relationship with the partner, concern about close people/family members being infected by COVID-19, and loneliness were all statistically associated with perceived stress [27]. A cross-sectional study based out of Cyprus examined the mental health of various healthcare workers during the pandemic [14]. Overall, almost 75% of PTs experienced moderate-to-high stress levels, 16% reported depression, and 12% reported having PTSD. Notably, compared to physicians (referent group), PTs' odds of experiencing stress, depression, or PTSD were not statistically different. In a mixed-method study examining work stress during the pandemic, researchers found PTs working with COVID-19 patients had poorer mental health outcomes than those who did not [33]. In the qualitative portion of the study, the authors highlight that lack of organizational support was a stressor.

Jácome et al. (2021) examined burnout among Portuguese PTs during the pandemic [28]. Results revealed that more than 40% of PTs experienced personal and work-related burnout, and 25% experienced patient-related burnout. Resilience, depression, and stress were predictors in the three burnout dimensions. Additionally, female PTs and working directly with COVID-19 patients increased the risk of burnout [28]. Similarly, in a study examining the sleep quality of Brazilian PTs, the researchers found that PTs working directly with patients had more severe symptoms of anxiety and stress than non-patient-facing PTs [29]. They also found that PPE shortage and fear of being infected were among their biggest fears. Finally, almost 90% of the sample exhibited poor sleep quality. A Polish study found that despite elevated scores in burnout, PTs had a statistically lower risk of burnout and reported higher job satisfaction than nurses [30]. In another Polish study, researchers found that PTs experienced burnout due to elevated scores in emotional exhaustion and depersonalization and lower scores in personal accomplishment [31]. Also, male PTs were at higher risk of experiencing burnout compared to their female counterpart. Furthermore, those with more than 20 years of work experience had higher scores in emotional exhaustion. Finally, PTs who did not engage in training programs had the highest burnout rates.

Ditwiler et al. (2021) conducted a qualitative study exploring the experiences of PTs regarding professional and ethical issues faced during the pandemic [34]. Results revealed that participants experienced confusion due to insufficient clinical evidence in terms of procedures during the pandemic. Also, participants reported stress, including moral distress due to physical and emotional barriers because of required isolation. Reportedly, improper use or lack of access to PPE was also a source of stress. Finally, due to the ongoing changes during the pandemic, participants note that managers' influence was problematic. In another qualitative study, similar results were found [35]. For instance, the moral distress concerning preventing family members from visiting dying patients was highlighted. PPE shortage was also a source of stress.

**Table 3.** Summary of studies examining the mental health of PTs during COVID-19 (n = 10). Note: See OTs summary for a study including OTs and PTs (n = 1).

First author, year	Study information	Results
Duarte, 2022 [27]	Country: Brazil Design: Cross-sectional Objective: Evaluate stress perception of stress during the pandemic.	- PTs reported increased perceived stress than before the pandemic. - Gender, age, reduction in family income, poor sleep, housework, relationship with the partner, concern about close people/family members being infected by COVID-19 and loneliness were all statistically associated with perceived higher stress.
*Chatzittofis, 2021 [14]	Country: Cyprus Design: Cross-sectional Objective: Examine mental health outcomes of healthcare workers during the pandemic	- 43% and 31% had medium and high stress, respectively. - 16% and 12% had clinical depression and PTSD symptoms, respectively. - Compared to physicians (referent group), PTs' odds of experiencing stress, depression, or PTSD were not statistically different.
Jácome, 2021 [28]	Country: Portugal Design: Cross-sectional Objective: To examine burnout among PTs working during the pandemic.	- 42% had personal, and work-related burnout, and 25% had patient related burnout. Resilience, depression and stress were predictors in the three burnout dimensions. - Gender (female) and working directly with patients were associated with burnout.
Lino, 2021 [29]	Country: Brazil Design: Cross-sectional Objective: To investigate the prevalence of sleep problems and associated factors among Brazilian PTs during the pandemic.	- 48%, 24% and 16% of frontline PTs feared being infected, PPE shortages and disease severity, respectively. - 86% were classified as poor sleepers -Frontline PTs had higher anxiety compared to non-frontline PTs.
*Szwamel, 2022 [30]	Country: Poland Design: Cross-sectional Objective: Examine correlated of burnout, anxiety, and depression among Polish healthcare workers during the pandemic.	- Compared to nurses, PTs had lower burnout and higher job satisfaction scores.
Pniak, 2021 [31]	Country: Poland Design: Cross-sectional Objective: Examine correlated of burnout, anxiety, and depression among Polish healthcare workers during the pandemic.	- PTs with over 20 years of work experience had higher emotional exhaustion scores. - Male PTs and PTs who rarely participated in training programs had higher burnout rates.
Yang, 2020 [32]	Country: South Korea Design: Cross-sectional Objective: Examine the mental health burden of COVID-19 on PTs.	- 32.3% had anxiety and 18.5% had depression. - PTs who lived with a dependent were more likely to have anxiety.
Hassem, 2022 [33]	Country: South Africa Design: Cross-sectional/mixed-method Objective: (1) Determine levels of mental and physical health, burnout, depression, anxiety and resilience and coping strategies used by South African PTs with and without exposure to patients with COVID-19. (2) Explore lived work experience, perceived health, and sources of support during the pandemic.	Quantitative: - Participants exposed to COVID-19 patients scored higher in mental health, anxiety and depression than those who were not exposed to COVID-19 patients. Qualitative: Both groups highlighted similar experiences and work-related challenges including lack of organizational support.
Ditwiler, 2021 [34]	Country: USA Design: Qualitative Objective: Explore the experiences of PTs regarding the professional and ethical issues they encountered during the COVID-19 pandemic.	- Uncertainty due to lack of COVID-specific clinical evidence and guidance causing confusion about procedures and care. - Frustration when assigned to work with COVID-19 patients and the burden of caring for COVID-19 patients, including physical and emotional “barriers” needed to protect against infection, were socially isolating and dehumanizing. - Stress, burnout, anxiety, fear, frustration, guilt, and moral distress were common among participants caused by the uncertainty of the pandemic. - The need for proper use of PPE and PPE shortage causing stress. - The influence of leaders and managers was problematic due to the ongoing changes due to the pandemic.
Palacios-Ceña, 2020 [35]	Country: Spain Design: Qualitative Objective: Explore the emotional experiences and feelings of PTs during the pandemic.	- Critical events such as death of a patient or a family member, hospitalizations of family members and dying along (moral distress). - PPE shortage. - Fear of infection and limited respirators.

*Note: *Several occupations, including PTs.

## 4. Discussion

Our rapid review examined the mental health of often neglected healthcare providers in the extant literature, such as MLPs and rehabilitation specialists, including OTs and PTs, during COVID-19. Our results demonstrated that, like other healthcare workers during the pandemic, MLPs, OTs, and PTs experienced various poor mental health outcomes, including anxiety, burnout, and depression. Overall, higher workload appeared to be a cogent theme among the three healthcare providers connected to poorer mental health outcomes [8],[21],[23],[25],[27],[33]. Many of the factors associated with poorer mental health outcomes, such as age [36], gender [2]–[4],[37], and having dependents [36], are similar to the results of other healthcare workers during COVID-19.

Our results revealed pandemic-specific factors that affected the mental health of healthcare workers that were not unique to a specific job role. The scarcity of PPE and fear of spreading the virus to a loved one due to direct work with COVID-19 or in a medical facility was a common theme in the identified studies [25],[27]–[29],[32]–[35]. While working with sick patients in a healthcare environment increases the risk of contagion, we suspect that COVID-19 fears might be somewhat alleviated due to better access to PPE, vaccination, and decreased mortality rates since the height of the pandemic. Moreover, our search revealed that improper communication or limited guidance on new treatments during the pandemic led to poorer mental health outcomes [25],[34]. This is unsurprising as research in pandemic preparedness suggests that all information about new knowledge and treatment must be communicated effectively within organizations during pandemic emergency responses [1]. This is an essential consideration for managerial personnel and policymakers.

Morally distressing events were among the pandemic-specific factors detected in our results, irrespective of the job role. For instance, PTs who worked directly with patients and their families experienced moral distress due to restrictions, disallowing visitors in medical settings [34],[35]. In a different context, MLPs felt obligated to work longer hours or work while feeling unwell, as they felt that in their absence, patient care would suffer [8]. These descriptions were outlined in the qualitative studies. Notably, we suspect that moral distress might be a factor in the cross-sectional studies we examined; however, survey-based questionnaires often limit detailed explanations for outcomes compared to qualitative studies.

While working predominantly in isolated environments such as laboratory settings with minimal-to-no interactions with patients or other healthcare providers, we suspect that MLPs might be experiencing unique challenges, including feeling forgotten and unappreciated. Due to the lack of research among MLPs, more research on this occupational group is required. Notably, our review did not uncover unique challenges faced by OTs and PTs, as the reported challenges were common among other frontline healthcare workers. However, this may be due to limited research among these professions.

Limited research among MLPs, OTs, and PTs during the pandemic has made it difficult to provide specific recommendations to address challenges that could be unique to their profession. Moreover, without further investigation and efforts to mitigate challenges faced by MLPs, OTs, and PTs, this could lead to more staff turnover, affecting service provision. Nevertheless, based on our findings, workload, and perceived social support may influence a healthcare worker's mental health, irrespective of the work type or the role of the pandemic [8],[14],[23],[25],[27],[28],[30],[31],[33]–[35]. While these factors might be difficult to manage, it is important to note that they are likely connected and working towards improving one might influence the other.

Evidence from a recent meta-analysis examining predictors of sickness absenteeism in nurses revealed that increased social support in the workplace statistically decreased the risk of sick leave [38]. Thus, we may deduce that a supportive work environment could have promising implications on one's mental health, reducing sick leave and, thus, improving employee retention. Unsurprisingly, improved staff retention would likely offer a more manageable workload for healthcare workers. To that end, we recommend to policymakers and managerial staff in healthcare settings focus on cultivating a positive work environment. This can be established by receiving training in leadership and team cohesion, allowing for improved communication among a healthcare team. Additionally, employees can feel supported by their management team through scheduled check-ins, and regular debriefing sessions, which is often dismissed in fast-paced environments [39]. Notably, scheduled meetings and debriefing sessions have favorable implications on service provision and team dynamics. Our recommendations are supported by the notion that employees seek meaning and respect in the workplace and thereby, need intrinsic incentives during the pandemic and beyond.

Coccia (2019) highlights the importance of implementing intrinsic incentives, especially in public organizations [40]. Compared to its extrinsic counterpart, which focuses on monetary gains, which could be fiscally challenging to offer in public sectors, intrinsic incentives within an organization focuses on the job itself and how it could provide personal satisfaction, including autonomy and empowerment. These incentive types, including personalized appreciation and timely communications, are associated with higher organizational job satisfaction and affective commitment. This approach could, in part, improve job retention in the healthcare sector as staff shortage [38],[39],[41] has been an issue that has been further exacerbated by the pandemic [42]–[45].

## 5. Limitations

The main limitation of this study is not conducting a quality assessment on the studies selected for the synthesis. To minimize the risk of including low-quality studies, only published papers in peer-reviewed journals were included, a common practice in rapid reviews. Therefore, results should be interpreted with some caution. Notwithstanding this limitation, there is an overall dearth of information for these occupational groups and thus, synthesizing this data highlights the need for more research.

## 6. Conclusions

This rapid review examined the mental health of MLPs, OTs, and PTs during the pandemic. Overall, our results suggest that MLPs might be experiencing additional challenges such as feeling unappreciated and forgotten within the healthcare sector due to the isolative nature of their work. OTs and PTs had similar factors associated with mental health, such as other healthcare workers. However, it is essential to note that with limited research on these healthcare groups before and during the pandemic, there could be unique factors about their jobs that are yet to be discovered. To improve retention and reduce staff shortages in healthcare, policymakers are encouraged to improve the workplace culture, embedding intrinsic incentives and better communication within the organization. Furthermore, more crisis preparation is required within organizations with a strong focus on providing timely and accurate information to their employees, including the newest treatments.
